# *De novo NFKBIA* variants within the N-terminal hotspot: consistent immunophenotype and divergent clinical presentations

**DOI:** 10.3389/fimmu.2026.1854185

**Published:** 2026-06-05

**Authors:** Rui Gan, Guangzhao Li, Lina Zhou, Li Wang, Rongxin Dai, Xuemei Tang, Junfeng Wu, Yanjun Jia, Qing Zhou, Xiaodong Zhao, Yunfei An

**Affiliations:** 1 Chongqing Key Laboratory of Child Rare Diseases in Infection and Immunity, Children’s Hospital of Chongqing Medical University, National Clinical Research Center for Children and Adolescents’ Health and Diseases, Ministry of Education Key Laboratory of Child Development and Disorders, Chongqing, China; 2 Department of Rheumatology and Immunology, Children’s Hospital of Chongqing Medical University, National Clinical Research Center for Children and Adolescents’ Health and Diseases, Ministry of Education Key Laboratory of Child Development and Disorders, Chongqing, China; 3 Department of Laboratory, Children’s Hospital of Chongqing Medical University, Chongqing, China; 4 Life Sciences Institute, Zhejiang University, Hangzhou, China; 5 Liangzhu Laboratory, Zhejiang University Medical Center, Hangzhou, China

**Keywords:** ectodermal dysplasia with immunodeficiency, inborn errors of immunity, IκBα, *NFKBIA*, NF-κB

## Abstract

**Background:**

Germline monoallelic gain-of-function (GOF) variants in *NFKBIA*, encoding IκBα, cause a rare immunodeficiency syndrome classically described as autosomal-dominant anhidrotic ectodermal dysplasia with immunodeficiency. However, the pathogenic spectrum of variants within the N-terminal hotspot and the extent to which distinct alleles converge on shared immunologic phenotypes are not fully defined.

**Methods:**

We studied four unrelated patients with *de novo* heterozygous *NFKBIA* variants (p.G33D, p.M37R, p.M37K, p.D31H), including two novel alleles (p.G33D and p.D31H). Clinical and immunological phenotyping, T-cell and B-cell subset analysis, and CFSE-based lymphocyte proliferation assays were performed. Functional consequences were assessed by TNF-α-induced IκBα degradation in patient PBMCs and by NF-κB dual-luciferase reporter assays in HEK293T cells expressing wild-type or mutant IκBα. A comprehensive literature review of all previously reported *NFKBIA* GOF cases was performed.

**Results:**

Clinical severity ranged from recurrent sinopulmonary infections onset in adolescence to severe infantile multisystem disease with bacterial, fungal, and opportunistic infections. All patients exhibited ectodermal abnormalities, and one had autoantibodies. Despite marked clinical heterogeneity, all four patients showed a qualitatively convergent lymphocyte phenotype characterized by expanded naïve T-cell and B-cell compartments and reduced memory and effector subsets. PHA-induced CD4^+^ and CD8^+^ T-cell proliferation was preserved in P1 and P3, whereas anti-CD3/CD28-induced T-cell proliferation, assessed only in P3, was impaired, while B-cell proliferation was preserved in the tested patients. Patient PBMCs exhibited markedly delayed or minimal TNF-α-induced IκBα degradation, and all four mutant proteins more strongly suppressed TNF-α-induced NF-κB reporter activity compared to wild-type IκBα. Baseline expression of the IκBα-EGFP fusion proteins was comparable across wild-type and all four mutant constructs.

**Conclusion:**

These findings broaden the clinical and genotypic spectrum of N-terminal IκBα GOF disease, identify a consistent immune phenotype characterized by expanded naïve and contracted memory lymphocyte compartments, and support defective regulated IκBα degradation and impaired lymphocyte maturation as shared features of N-terminal IκBα GOF disease.

## Introduction

The canonical NF-κB pathway is essential for host defense and ectodermal development (1). In resting cells, IκBα, encoded by *NFKBIA*, retains NF-κB dimers such as p65/RelA and p50 in the cytoplasm. Upon stimulation, IκBα is phosphorylated at serine 32 and serine 36, leading to its ubiquitination and proteasomal degradation, which enables NF-κB nuclear translocation and transcription of genes involved in innate and adaptive immunity (1, 2). Germline monoallelic gain-of-function (GOF) variants in IκBα impair this degradation step and typically cause autosomal-dominant anhidrotic ectodermal dysplasia with immunodeficiency (AD EDA-ID), although the disease spectrum is broader than the classical syndromic definition (3, 4).

Since the first report in 2003 (5), the allelic spectrum has expanded from recurrent N-terminal missense substitutions affecting the phosphodegron or adjacent residues, such as D31N, S32I, S32G, S32R, S32N, S32C, G33V, L34P, S36Y, S36A, S36P, M37K, and M37R (3, 5–19), to truncating variants like Q9X, W11X, E14X (20–25), and the recently described Q228X (19). Patients exhibit significant clinical heterogeneity, including recurrent bacterial, mycobacterial, fungal, and viral infections, variable humoral and cellular immune defects, autoinflammation, and varying ectodermal abnormalities (3, 16, 19). Limited genotype-phenotype correlation studies indicate that N-terminal missense variants generally result in more severe impairment than truncating alleles (13).

However, the pathogenic spectrum of newly identified variants within the D31–M37 hotspot and the extent to which distinct alleles converge on shared immunologic phenotypes remain poorly defined. In this study, we report four unrelated patients with *de novo* heterozygous *NFKBIA* variants, including two novel missense alleles, p.G33D and p.D31H. We further characterize the clinical and molecular spectrum of N-terminal IκBα GOF disease and identify a consistent immunologic phenotype marked by naïve-skewed lymphocyte compartments, despite significant clinical heterogeneity.

## Materials and methods

### Patients

Four unrelated patients with heterozygous *NFKBIA* variants were enrolled in this study. Clinical data were collected at the time of first evaluation and updated during follow-up. Peripheral blood samples from patients and healthy controls were obtained for immunologic and functional studies. The study was approved by the Institutional Review Board of Children’s Hospital of Chongqing Medical University (No. 2024-22), and written informed consent was obtained from the legal guardians of all patients. None of the four patients included in the present cohort has been previously reported.

### Literature review and case summary

Previously reported patients with pathogenic NFKBIA GOF variants were identified from the published literature and from the reference lists of relevant review articles and case reports. Clinical, genetic, immunologic, treatment, and outcome data were extracted when available and summarized in **Supplementary Tables 2** and **S3**. Because of heterogeneous reporting across published cases, missing or unavailable features were not imputed.

### Genetic analysis and Sanger sequencing

Whole-exome sequencing was performed for molecular diagnosis. Candidate *NFKBIA* variants were validated by Sanger sequencing in the probands and their parents. Genomic DNA was extracted from peripheral blood with the TGuide S32 kit (Tiangen). The variant-containing region was amplified using LA Taq DNA polymerase with GC buffer I (Takara) with primers 5′-CGCCCCAGCGAGGAAGCAG-3′ and 5′-CCTCCGCCACTTACGAGTC-3′ under standard PCR conditions (94 °C for 1 min; 30 cycles of 94 °C for 30 s, 61 °C for 30 s, and 72 °C for 2 min; final extension at 72 °C for 5 min). PCR products were subjected to Sanger sequencing (Sangon Biotech). Conservation of the affected residues was assessed by multispecies sequence alignment.

### Structural modeling of NFKBIA variants

The predicted structure of wild-type human IκBα was obtained from the AlphaFold Protein Structure Database (AF-P25963). Missense variants were introduced in silico, and the corresponding mutant structures were predicted using AlphaFold2. Structural visualization and figure preparation were performed in PyMOL (version 3.03), with analysis focused on local conformational changes and predicted polar contacts in the N-terminal phosphodegron region surrounding Ser32 and Ser36. These models were used only for exploratory visualization of possible local contact-network changes.

### Isolation of peripheral blood mononuclear cells

PBMCs were isolated from fresh peripheral blood by density-gradient centrifugation. Blood was diluted 1:1 with PBS, layered onto lymphocyte separation medium, and centrifuged at 800 × g for 20 min at room temperature. The PBMC layer was collected and washed with PBS. Cells were used immediately or cryopreserved in 90% FBS and 10% DMSO and stored in liquid nitrogen.

### Immunophenotyping of T and B subsets

**Figure 1:**
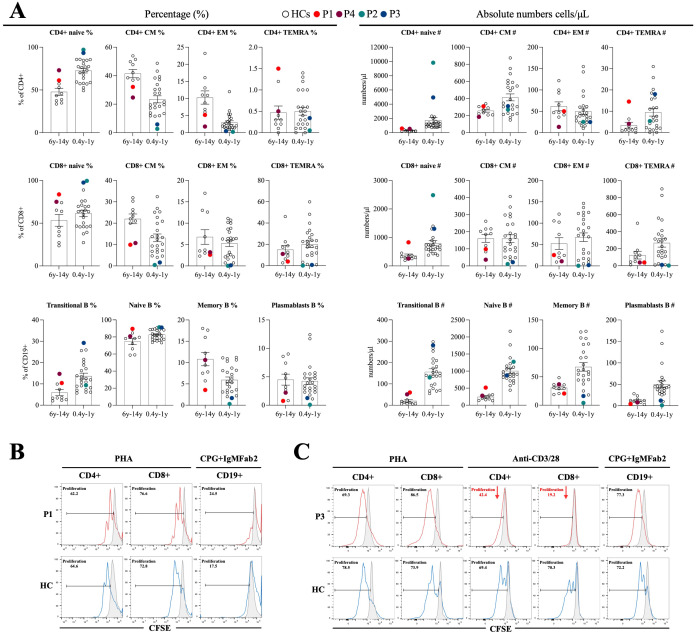
Naïve-skewed T/B phenotype and lymphocyte proliferation assays in patients with *NFKBIA* variants. **(A)** Flow-cytometric analysis of peripheral blood T-cell and B-cell subsets in the four patients and healthy controls. Percentages (left) and absolute numbers (right) of CD4+ and CD8+ T-cell subsets and CD19+ B-cell subsets are shown. analyzecolorGray circles indicate individual locally recruited healthy controls (6–14 y, n = 8; 0.4–1 y, n = 22) analyzed in parallel with patient samples. P1 and P4 were analyzed at 14 years 9 months and 6 years 1 month, respectively, and were compared with the 6–14-year healthy control group; P2 and P3 were analyzed at 5 months and 4 months, respectively, and were compared with the 0.4–1-year healthy control group. Colored symbols indicate individual patients; bars show mean ± SEM of the healthy control groups. CM, central memory; EM, effector memory; TEMRA, terminal effector memory T cells re-expressing CD45RA. **(B)** Representative CFSE dilution histograms from P1 and a healthy control after 72 h of stimulation with PHA for T-cell proliferation and CpG plus anti-human IgM F(ab′)2 for B-cell proliferation. **(C)** Representative CFSE dilution histograms from P3 and a healthy control after 72 h of stimulation with PHA, anti-CD3/CD28, or CpG plus anti-human IgM F(ab′)2, as indicated. Anti-CD3/CD28-induced proliferation was assessed only in P3 because of limited patient PBMC availability. The percentage of proliferating cells is shown in each plot.

Flow cytometry was performed on peripheral blood to characterize T-cell and B-cell subsets, as previously described (26). For T-cell phenotyping, 50 μL of whole blood was stained with antibodies against TCRαβ, TCRγδ, CD3, CD4, CD8, CD27, and CD45RA. For B-cell phenotyping, 50 μL of whole blood was stained with antibodies against CD19, CD38, CD24, IgD, and CD27. After incubation for 30 min at room temperature in the dark, red blood cells were lysed with 2 mL lysis buffer for 6 min at 37 °C. Cells were then washed with PBS and acquired by flow cytometry. Healthy controls were analyzed in two age-stratified groups (6–14 y, n = 8; and 0.4–1 y, n = 22), according to patients’ ages at the time of immunophenotyping. P1 (14 years 9 months) and P4 (6 years 1 month at immunophenotyping) were compared with the 6–14-year healthy control group; P2 (5 months) and P3 (4 months) were compared with the 0.4–1-year healthy control group. All healthy controls were recruited locally and analyzed in the same experimental batch as the respective patient samples, using identical flow cytometry panels and staining conditions. Data are presented as percentages and absolute numbers. For **Supplementary Table 1**, patient lymphocyte subset values were interpreted using published age-stratified Chinese pediatric reference intervals from Ding et al. (26), which were selected because all four patients were Chinese and these values provide population-appropriate normative comparators for pediatric lymphocyte subset assessment. These published reference values were not used to generate the healthy control data points in **Figure 1A**.

### CFSE-based lymphocyte proliferation assay

PBMCs from patients and healthy controls were labeled with CFSE and cultured for 72 h under unstimulated conditions or with PHA (5 μg/mL), plate-bound anti-CD3/CD28 (5 μg/mL), or CpG (1 μM) plus anti-human IgM F(ab′)2 (13 μg/mL). Cells were then stained with antibodies against CD4, CD8, and CD19, and proliferation of CD4+ T-cells, CD8+ T-cells, and CD19+ B-cells was assessed by CFSE dilution by flow cytometry.

### TNF-α–induced IκBα degradation and immunoblotting

PBMCs from patients and healthy controls were stimulated with 20 ng/mL TNF-α (PeproTech AF-300-01A) for the indicated times, lysed, and subjected to SDS-PAGE and immunoblotting. Antibodies used were anti-IκBα (Cell Signaling Technology, #4812, 1:1000), anti-vinculin (Proteintech, #66305-1-Ig, 1:1000), anti-α-tubulin (Proteintech, #11224-1-AP, 1:2000), anti-GAPDH-HRP (Proteintech, #HRP-60004, 1:10,000), and anti-β-actin-HRP (Proteintech, #HRP-66009, 1:10,000), with HRP-conjugated goat anti-rabbit and goat anti-mouse secondary antibodies (Cell Signaling Technology, #7074 and #7076, both 1:10,000). IκBα band intensity was normalized to the corresponding loading control (vinculin, α-tubulin, GAPDH, or β-actin, as indicated in each panel), and relative expression was calculated as the ratio of IκBα to loading control at each time point. The four patients were assessed at different clinical time points; each patient’s experiment was therefore conducted independently, with a concurrently analyzed healthy control in the same batch, using an antibody panel optimized for that batch.

### NF-κB dual-luciferase reporter assay

HEK293T cells were seeded into 96-well plates at 2–3 × 10^4^ cells per well 18–24 h before transfection. Cells were transiently co-transfected with an NF-κB firefly luciferase reporter plasmid (10 ng; Beyotime, D2207), a Renilla luciferase control plasmid (2 ng; Beyotime, D2760), and expression constructs pIκB-EGFP (BD Biosciences Clontech, 6919-1) encoding wild-type or mutant IκBα (40 ng per well) using PEI. 18 h after transfection, cells were left unstimulated or were stimulated with TNF-α (20 ng/mL) for another 18 h, and luciferase activity was measured using the Dual-Luciferase Reporter Assay System (Promega, E1960). Firefly luciferase activity was normalized to Renilla luciferase activity. In parallel, HEK293T cells were transfected with the same wild-type or mutant pIκB-EGFP constructs (40 ng per well) under identical conditions, and whole-cell lysates were analyzed by immunoblotting with an anti-IκBα antibody (Cell Signaling Technology, #4812, 1:1000) and anti-GAPDH-HRP (Proteintech, #HRP-60004, 1:10,000) to confirm comparable baseline expression of the WT and mutant IκBα-EGFP fusion proteins.

### Statistical analysis

Data are shown as mean ± SEM where applicable. For luciferase assays, data were analyzed by two-way ANOVA with Tukey’s multiple-comparisons test. A two-sided P value < 0.05 was considered statistically significant. Lymphocyte subset comparisons and IκBα densitometry are presented as descriptive analyses; no formal group statistical testing was performed for these data, given the small cohort size and the descriptive intent of these analyses. The two-way ANOVA applies exclusively to the NF-κB dual-luciferase reporter assay.

## Results

### Clinical and genetic features of patients with *de novo NFKBIA* variants

**Figure 2:**
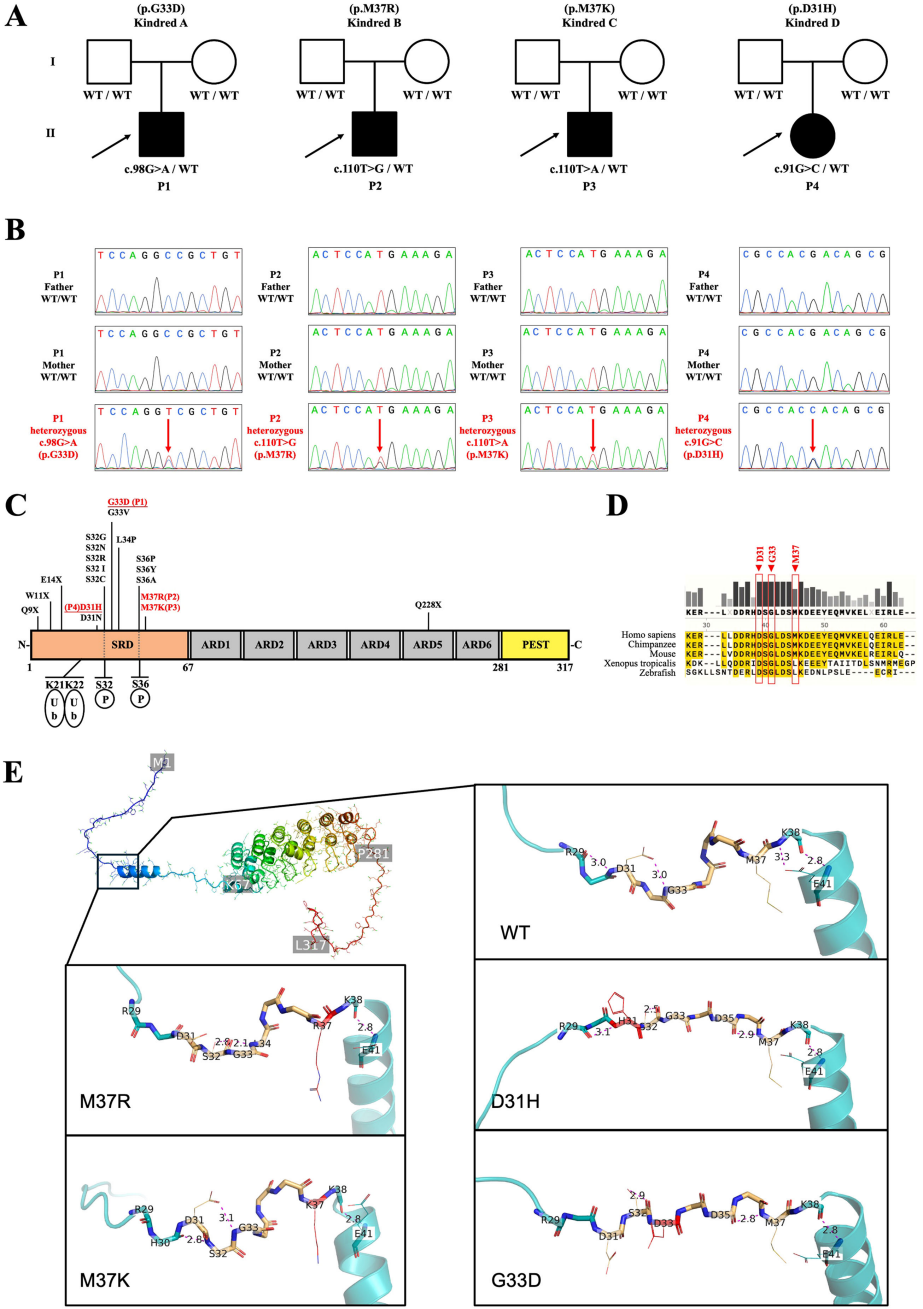
Identification of four *de novo NFKBIA* variants. **(A)** Pedigrees of the four unrelated kindreds. Black symbols indicate affected individuals. **(B)** Sanger sequencing confirming the heterozygous *NFKBIA* variants in the probands and wild-type sequences in both parents. Arrows indicate the mutant nucleotide in each proband. **(C)** Schematic representation of IκBα and the location of previously reported variants together with the variants identified in this study, highlighted in red. SRD, signal response domain; ARD, ankyrin repeat domain; PEST, proline/glutamic acid/serine/threonine-rich domain. Lys21 and Lys22 indicate ubiquitination sites, and Ser32 and Ser36 indicate phosphorylation sites required for regulated IκBα degradation. **(D)** Multispecies protein sequence alignment showing evolutionary conservation of the affected residues within the N-terminal hotspot of IκBα. **(E)** Structural modeling of *NFKBIA* variants. AlphaFold2-predicted full-length structure of human IκBα, with the N-terminal signal-receiving region boxed. Local structural model of wild-type and mutant IκBα centered on residues 29–41. Residues of interest are shown as sticks and labeled individually. The protein backbone is shown as cartoon representation. Magenta dashed lines indicate predicted polar contacts/hydrogen bonds, and selected interatomic distances are shown in angstroms. These AlphaFold2-based mutant models are presented as structural hypotheses to illustrate possible local contact-network changes around the phosphodegron and should not be interpreted as experimentally determined structures or as standalone evidence of pathogenicity.

We studied four unrelated patients with *de novo* heterozygous *NFKBIA* variants (**Figure 2A**; **Table 1**). P1 presented at 12 years of age with recurrent fever, pneumococcal pneumonia, otitis media, chronic bronchitis, and bronchiectasis, together with irregular dentition and conical teeth. He started regular intravenous immunoglobulin (IVIG) replacement and prophylactic antibiotics at 14 years and 10 months of age and remained clinically stable. P2 presented in the first month of life with recurrent diarrhea with hematochezia, anemia, fever, and chronic mucocutaneous candidiasis, followed by local BCGitis and infections caused by multiple pathogens. He had sparse hair, hypodontia, conical teeth, reduced serum IgG, and positive autoantibodies, and died of acute respiratory distress syndrome at 9 months of age despite antimicrobial therapy and regular IVIG. P3 developed local BCGitis at 2 months of age, followed by recurrent infections and anemia. He had hypodontia, abnormal dental development, markedly reduced serum IgA, and elevated IgM (3.61 g/L; reference range, 0.43–1.63 g/L), and underwent successful hematopoietic stem cell transplantation (HSCT) at 1.5 years of age. P4 also developed local BCGitis at 2 months of age, later followed by recurrent pneumonia, fever, and anemia. She had conical teeth and abnormal dental development and remained clinically stable on IVIG and prophylactic antibiotics.

**Table 1:** Genetic and clinical features of patients with heterozygous *NFKBIA* variants.

Individual	P1	P2	P3	P4
Genotype	c.98G>A	c.110T>G	c.110T>A	c.91G>C
IκBα protein	p.G33D	p.M37R	p.M37K	p.D31H
Inheritance	*de novo*	*de novo*	*de novo*	*de novo*
Zygosity	heterozygous	heterozygous	heterozygous	heterozygous
Demographics				
Ethnicity	Chinese	Chinese	Chinese	Chinese
Sex	Male	Male	Male	Female
Age at onset	12y	1m	2m	2m
Age at diagnosis	14y8m	4m26d	2m28d	5y6m
EDA features				
	irregular dentition and conical teeth	sparse hair, hypodontia and conical teeth	hypodontia	irregular dentition and conical teeth
Infections				
	*Streptococcus pneumoniae* pneumonia, otitis media	Local BCGitis, *Pseudomonas aeruginosa*, *Pneumocystis jirovecii* pneumonia, CMC	Local BCGitis, *Streptococcus pneumoniae* pneumonia, rotavirus, CMC	Local BCGitis, pneumonia
Immunological findings				
IgG (g/L)	10.2 (RR 5.280-21.900)	2.26 (RR 2.860-16.800)	8.56 (RR 2.860-16.800)	14.5 (RR 7-16.0)
IgA (g/L)	3.05 (RR 0.440-4.410)	0.139 (RR 0.10-1.290)	<0.0667 (RR 0.190-1.750)	0.86 (RR 0.7-4.0)
IgM (g/L)	0.809 (RR 0.48-2.26)	1.62 (RR 0.21-1.92)	3.61 (RR 0.43-1.63)	2.14 (RR 0.4-2.3)
IgE (IU/mL)	5.1 (RR 0-165.0)	<5.0 (RR 0-165.0)	<5.0 (RR 0-165.0)	15.3 (RR 0-100)
C3 (g/L)	0.91 (RR 0.7-2.06)	0.41 (RR 0.79-1.79)	1.19 (RR 0.7-2.06)	1.18 (RR 0.7-2.06)
C4 (g/L)	0.14 (RR 0.11-0.61)	0.09 (RR 0.11-0.61)	0.32 (RR 0.11-0.61)	0.18 (RR 0.11-0.61)
Autoantibodies	Negative	TGAb (+), anti-TPO Ab (+), anti-Ro52 Ab (+)	Negative	Negative
Treatment and outcome				
	IVIG, antibiotics. Clinically stable	IVIG, antibiotics and antimycobacterial regimen. Died of ARDS at 9 months	IVIG, antibiotics and antimycobacterial regimen. HSCT at 1.5 years	IVIG, antibiotics and antimycobacterial regimen. Clinically stable

CMC, chronic mucocutaneous candidiasis; BCG, Bacillus Calmette-Guérin; RR, Reference Range; IVIG, intravenous immunoglobulin; HSCT, hematopoietic stem cell transplantation Serum immunoglobulin levels shown in **Table 1** were measured before the initiation of IVIG replacement therapy.

Whole-exome sequencing identified four heterozygous *NFKBIA* variants: c.98G>A (p.G33D) in P1, c.110T>G (p.M37R) in P2, c.110T>A (p.M37K) in P3, and c.91G>C (p.D31H) in P4. All variants were confirmed by Sanger sequencing (**Figure 2B**). The M37K and M37R variants have been reported previously (8, 9), whereas p.G33D and p.D31H are novel. The affected residues are highly conserved across species (**Figures 2C, D**).

Structural modeling placed p.D31H, p.G33D, p.M37R, and p.M37K within or immediately adjacent to the conserved N-terminal phosphodegron of IκBα surrounding Ser32 and Ser36, the two inducible phosphorylation sites required for subsequent ubiquitination and proteasomal degradation. Compared with wild-type IκBα, all four variants were predicted to alter the local contact network around this short motif (**Figure 2E**). p.D31H replaces the acidic Asp31 residue immediately N-terminal to Ser32 with histidine, causing loss of the native negative charge at Asp31, with potential pH-dependent protonation of His31 adjacent to Ser32, thereby altering the local charge and polar environment adjacent to the first phospho-acceptor site. p.G33D replaces a structurally flexible glycine within the motif core with a negatively charged aspartate and was predicted to produce the most apparent local rearrangement, which may perturb the spatial presentation of Ser32 and Ser36. p.M37R and p.M37K replace the neutral Met37 residue immediately C-terminal to Ser36 with positively charged side chains, altering the predicted contact pattern around Lys38 and the downstream helical segment. These local changes may reduce the efficiency with which the N-terminal phosphodegron is recognized or positioned for signal-induced phosphorylation by the IKK complex.

### Naïve-skewed T/B phenotype and T-cell proliferation defect

Previous reports on IκBα GOF disease have described heterogeneous but recurrent lymphocyte abnormalities, including lymphocytosis, T-cell dysfunction, increased naïve T-cell predominance with reduced antigen-experienced CD8+ subsets in some patients, and decreased memory B-cell compartment (3, 5, 6, 25). We performed multiparameter immunophenotyping of peripheral T-cell and B-cell subsets in all four patients, using age-matched controls and analyzing them in two age-stratified groups (**Figure 1A**; **Supplementary Table 1**). Despite clinical heterogeneity, all four patients exhibited a convergent lymphocyte phenotype. Both CD4+ and CD8+ compartments showed increased naïve T-cells and reduced central memory, effector memory, and TEMRA subsets, particularly in the CD8+ lineage. A parallel shift was observed in the B-cell compartment, with expanded transitional and naïve B-cells and reduced memory B-cells and plasmablasts. Several patient-specific quantitative variations were noted on this shared background: P2 and P3 showed absolute lymphocytosis driven primarily by naïve CD4+ T-cell expansion; P3 additionally showed an elevated absolute NK-cell count; and P4 showed an increased γδ T-cell fraction together with modestly reduced absolute total and CD8+ T-cell counts. The naïve-predominant, memory-contracted distribution within both the CD4+ and CD8+ compartments was nonetheless consistently observed across all four patients (**Figure 1A**; **Supplementary Table 1**).

We assessed lymphocyte proliferative responses in patients with available PBMCs (**Figures 1B, C**). Under PHA stimulation, CD4+ and CD8+ T-cell proliferation was preserved in P1 and P3. Anti-CD3/CD28 stimulation could be assessed only in P3 because of limited patient material. In this patient, both CD4+ and CD8+ T-cell proliferation were markedly reduced after anti-CD3/CD28 stimulation compared with the healthy control tested in parallel. CD19+ B-cell proliferation induced by CpG plus anti-human IgM F(ab′)2 was preserved in both P1 and P3. These findings indicate preserved PHA-induced T-cell proliferation in the two tested patients and impaired anti-CD3/CD28-induced T-cell proliferation in P3, but they do not establish a cohort-wide anti-CD3/CD28 proliferation defect.

### Patient-derived *NFKBIA* variants impair inducible IκBα degradation and exhibit gain-of-function properties

**Figure 3:**
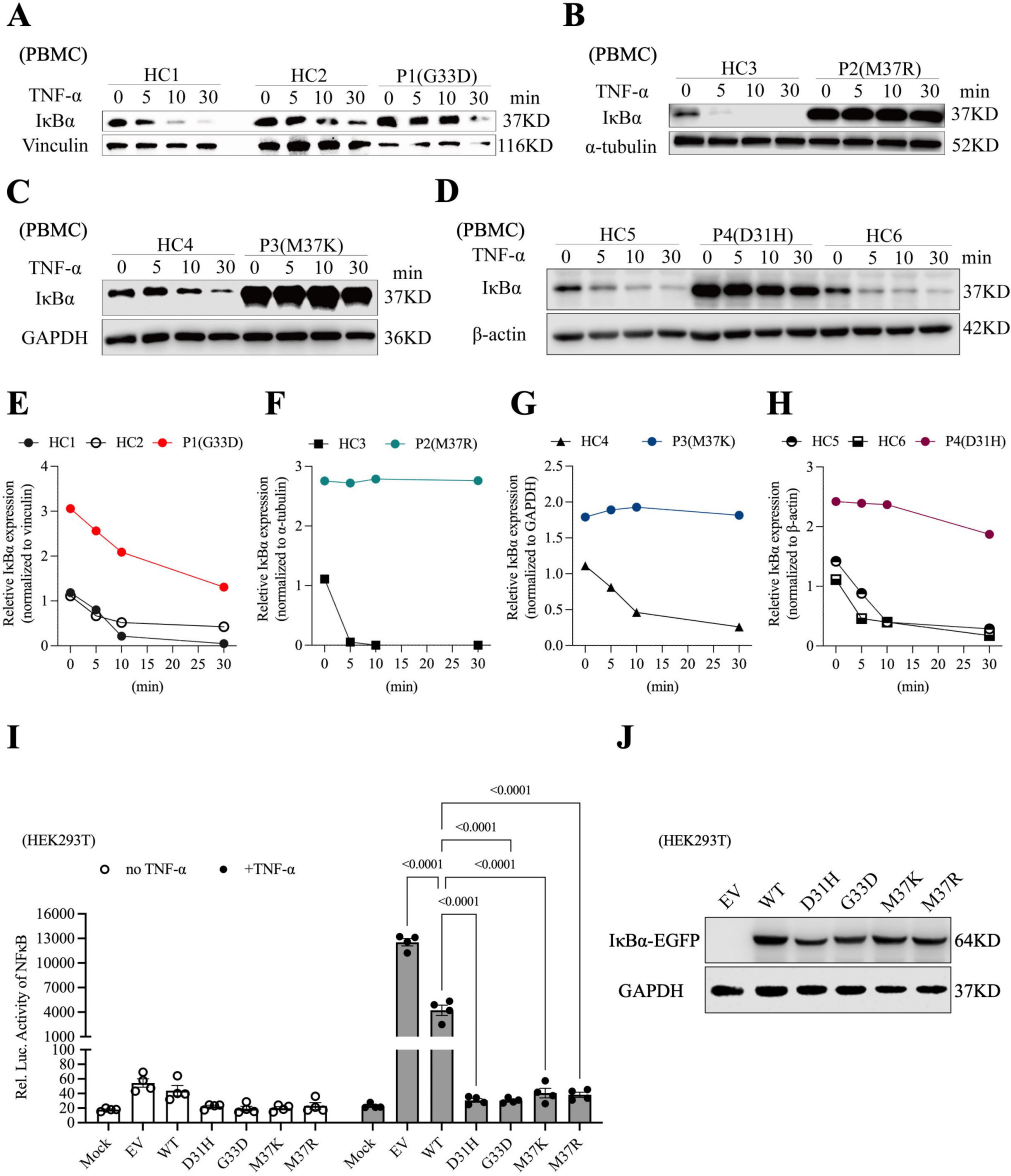
Patient-derived *NFKBIA* variants impair inducible IκBα degradation and suppress TNF-α–induced NF-κB reporter activity. **(A–D)** Immunoblot analysis of IκBα degradation in PBMCs from patients and healthy controls after TNF-α stimulation for the indicated times (0, 5, 10, and 30 min). Loading controls were vinculin in **(A)**, α-tubulin in **(B)**, GAPDH in **(C)**, and β-actin in **(D)**. **(E–H)** Densitometric quantification of IκBα protein abundance in the experiments shown in **(A–D)**, normalized to the corresponding loading control. **(I)** NF-κB dual-luciferase reporter assay in HEK293T cells transiently expressing wild-type or mutant IκBα constructs. Cells were left unstimulated or stimulated with TNF-α, as indicated. Firefly luciferase activity was normalized to Renilla luciferase activity. Data are shown as mean ± SEM. P values were calculated by two-way ANOVA with Tukey’s multiple-comparisons test. EV, empty vector. **(J)** Representative immunoblot showing baseline expression of IκBα-EGFP in HEK293T cells transiently transfected with empty vector (EV), wild-type (WT), or mutant IκBα-EGFP constructs (p.D31H, p.G33D, p.M37K, p.M37R) under the same transfection conditions as in **(I)**. GAPDH was used as the loading control.

To evaluate the functional consequences of these variants, we stimulated peripheral blood mononuclear cells (PBMCs) with TNF-α and examined IκBα degradation by immunoblotting (**Figures 3A–H**). In healthy controls, IκBα was rapidly degraded and became almost undetectable within 5–10 minutes. In contrast, PBMCs from patients P1–P4 showed markedly delayed or minimal degradation, with persistently higher IκBα levels throughout the 30-minute stimulation period.

Next, we assessed NF-κB activity in HEK293T cells overexpressing wild-type or mutant IκBα using a dual-luciferase reporter assay (**Figure 3I**). All four mutant proteins more effectively suppressed TNF-α-induced NF-κB activity compared to wild-type IκBα. Baseline expression of the IκBα-EGFP fusion protein was comparable across WT and all four mutant constructs (**Figure 3J**), indicating that the enhanced inhibitory activity of the mutants reflects an intrinsic functional difference rather than differences in protein abundance. Collectively, the degradation assays in patient cells and heterologous reporter assays establish p.G33D, p.M37R, p.M37K, and p.D31H as GOF alleles and support defective regulated IκBα degradation as the primary molecular abnormality in this cohort.

## Discussion

We report four unrelated patients with *de novo* heterozygous IκBα variants, including two novel missense alleles, p.G33D and p.D31H. Despite marked clinical heterogeneity, all patients shared a common biologic framework characterized by ectodermal abnormalities, infection susceptibility, a naïve-skewed lymphocyte phenotype, and defective inducible IκBα degradation. These findings expand the pathogenic spectrum of N-terminal *NFKBIA* variants and further define the immunologic phenotype of IκBα GOF disease.

With the addition of these four patients, the total number of reported patients with IκBα GOF disease now stands at 33, including one mosaic carrier (3, 12, 14–19, 23–25). This update provides a comprehensive overview of the clinical, genetic, and molecular spectrum of the disease (**Supplementary Tables 2**, **3**). Our cohort aligns with the established framework of IκBα GOF disease. While germline IκBα GOF was initially recognized as classical autosomal dominant ectodermal dysplasia with immunodeficiency (EDA-ID), subsequent reports have broadened the phenotype to include immunodeficiency without overt ectodermal dysplasia and, in some alleles, more inflammation-predominant presentations (10, 14, 19). All four variants identified here are missense mutations within the critical D31–M37 region, reinforcing this segment as a mutational hotspot.

Across all 33 reported patients, the clinical spectrum of *NFKBIA* GOF disease exhibits substantial heterogeneity but also several consistent themes that become clearer when cases are stratified by variant class (**Supplementary Table 2**; **Supplementary Figure 1**). Immunodeficiency, operationally defined by susceptibility to recurrent or severe infections, was documented in 30 of 33 patients (91%), with the three patients lacking this feature all carrying the C-terminal Q228X allele. Ectodermal dysplasia was present in 24 of the 32 patients with available data (75%); the frequency was 78% (18/23 patients with reportable data) among N-terminal missense carriers in the phosphodegron region, 100% (6/6) among carriers of N-terminal truncating alleles (W11X, Q9X, E14X), and 0% (0/3) among Q228X patients. All three Q228X patients presented with isolated rheumatologic autoinflammation without ectodermal involvement or recurrent infection, consistent with the distinct pathomechanism discussed below (19). The infectious spectrum among the 30 N-terminal variant carriers was broad: bacterial infections were nearly universal; mycobacterial complications were documented in approximately 8 of 30 N-terminal carriers (27%); and fungal infections including chronic mucocutaneous candidiasis were present in 11 of 30 (37%). These quantitative patterns are summarized visually in **Supplementary Figure 1**.

However, the clinical heterogeneity, ranging from adolescent-onset sinopulmonary infection in P1 to severe infantile multisystem disease in P2, suggests that variant position alone does not fully account for phenotypic severity. The point is reinforced by the M37R/M37K comparison within our cohort: P2 and P3 carried distinct substitutions at the same Met37 residue, showed predicted local rearrangements of the phosphodegron (**Figure 2E**), and exhibited similarly impaired TNF-α-induced IκBα degradation and enhanced inhibition of NF-κB reporter activity, yet differed dramatically in clinical outcome. This intra-cohort contrast is further supported by historical reports, in which the p.M37R patient of Giancane et al. died before scheduled HSCT (9), while the p.M37K patient of Schimke et al. survived to receive HSCT (8). Furthermore, identical *NFKBIA* GOF alleles have been associated with markedly divergent phenotypes within the same family, as exemplified by the three members of a Turkish kindred with p.S36A reported by Sogkas et al., in whom severity ranged from severe juvenile rheumatoid arthritis with CNS tuberculosis in the father to mild recurrent respiratory infection in the younger daughters (14). Several non-mutually exclusive modifiers may contribute to this expressivity, including the timing and microbial burden of early-life infectious exposure, age at diagnosis and time-to-initiation of prophylactic treatment, and contributions of non-hematopoietic *NFKBIA* deficiency, as suggested by the failure of wild-type bone-marrow transfer to rescue lymphoid organogenesis in the *Nfkbia*^WT/S32I^ knock-in mouse (4). Importantly, despite this clinical heterogeneity, the consistent lymphocytic signature, characterized by naïve-skewed T- and B-cell compartments with contraction of antigen-experienced subsets, was identifiable in P1 to P4, suggesting that the cellular consequence of impaired regulated IκBα degradation is detectable across the severity spectrum and may serve as a diagnostic clue even in patients with later, more indolent presentations.

A key finding of this study is the qualitatively reproducible lymphocyte-maturation phenotype shared by all four patients, namely a directional expansion of naïve T-cell and B-cell compartments with concomitant contraction of memory, effector, and terminally differentiated subsets. This maturation-stage shift indicates that a central consequence of IκBα GOF is impaired maturation to antigen-experienced states. The proliferation data should be interpreted cautiously. PHA-induced CD4+ and CD8+ T-cell proliferation was preserved in P1 and P3, whereas impaired anti-CD3/CD28-induced T-cell proliferation was observed in P3. This result therefore suggests a possible stimulus-dependent T-cell functional defect in P3 rather than a conclusion that can be generalized to the entire cohort, consistent with prior reports showing variable T-cell proliferative responses among patients with *NFKBIA* GOF variants. Further studies in additional patients will be required to determine whether impaired TCR/CD28-mediated proliferation is a recurrent feature of specific *NFKBIA* genotypes or reflects patient-specific immune status, age, treatment, or disease activity. P3 also showed elevated serum IgM together with marked IgA deficiency, representing a hyper-IgM-like dysgammaglobulinemia rather than isolated immunoglobulin abnormality. Similar IgM-predominant or dysgammaglobulinemic patterns have been reported in patients with *NFKBIA* GOF variants, including cases with impaired memory or switched-memory B-cell compartments (3, 8, 17). In the context of the markedly reduced memory B-cell and plasmablast compartments observed in our cohort, this finding further supports a broader defect in humoral maturation. However, because CD40/CD40L-dependent signaling, class-switch recombination, and somatic hypermutation were not directly assessed in P3, the mechanism underlying this IgM-predominant pattern remains to be defined.

The infectious phenotype in our cohort expands the practical clinical indicators for diagnosis. Notably, three patients (P2–P4) developed local BCGitis early in infancy. This pattern is consistent with previous reports of BCG-related disease and other mycobacterial complications in patients with *NFKBIA* variants (7, 10, 11, 16, 24). Therefore, IκBα GOF disease should be considered in infants presenting with early BCGitis or other BCG-related complications, particularly in BCG-vaccinating countries and when accompanied by ectodermal abnormalities, chronic mucocutaneous candidiasis, or a naïve-skewed T- and B-cell phenotype with reduced memory compartments. In addition, the later onset in P1 indicates that IκBα GOF disease is not restricted to infancy and may remain undiagnosed until later childhood.

Our findings also have therapeutic implications. IVIG, antimicrobial prophylaxis, and aggressive infection control remain essential and may stabilize selected patients. HSCT has been attempted in a substantial proportion of reported patients with *NFKBIA* GOF disease, but outcomes have been heterogeneous (**Supplementary Table 2**) (3, 27–30). In our updated summary, at least 15 of 33 reported patients underwent HSCT. Long-term benefit has been documented in selected cases, including durable full donor chimerism after HSCT in the first reported p.S32I patient and stable mixed donor chimerism after reduced-intensity conditioning in a patient with p.Q9X (27, 28). However, several patients died after HSCT or during the peri-transplant period, including patients with severe N-terminal missense variants, with reported complications including infection, hemorrhage, graft instability, or severe pre-existing organ involvement (3, 27–30). These variable outcomes suggest that HSCT can improve the hematopoietic immune defect in some patients but may not fully reverse established organ damage or developmental manifestations involving non-hematopoietic or stromal compartments. In our cohort, one patient died in infancy before potential HSCT, one underwent HSCT with a favorable early outcome at last follow-up, and two remain clinically stable on supportive therapy. Therefore, the optimal timing, conditioning regimen, and candidate selection for HSCT in *NFKBIA* GOF disease remain unresolved and require further longitudinal follow-up.

It is important to distinguish the molecular phenotype of N-terminal hotspot variants from that of the recently described Q228X variant, which causes a C-terminal truncation of IκBα. Unlike N-terminal variants, Q228X does not impair stimulus-induced IκBα degradation, but instead reduces nuclear translocation of NF-κB1 p50 in a manner resembling functional NF-κB1 haploinsufficiency. Clinically, Q228X presents with autoinflammatory arthritis and psoriasis rather than combined immunodeficiency and ectodermal dysplasia, and is associated with enhanced inflammasome activation. Notably, Q228X patients also display reduced switched memory B cells, suggesting that impaired B cell maturation may represent a shared immunophenotypic consequence of disturbed canonical NF-κB activity, regardless of the specific upstream mechanism. These mechanistic and clinical distinctions underscore that *NFKBIA* GOF is not a uniform disease entity, and that genotype-mechanism-phenotype stratification will be essential for future diagnostic and therapeutic frameworks.

This study has several limitations. Given that the disease is rare, the cohort size is relatively small. Furthermore, not all functional assays could be performed in all patients due to limited patient-derived material. In particular, we did not perform NF-κB nuclear translocation assays or IL-1R/TLR stimulation assays in patient-derived cells; IκBα degradation experiments were performed as single experiments per patient, as P2 is deceased, P3 has undergone HSCT, and material from P1 and P4 was exhausted during initial analyses. Therefore, the mechanistic conclusions of this study are primarily based on TNF-α-induced IκBα degradation in PBMCs and TNF-α-induced NF-κB reporter assays in a heterologous expression system. Although these data support impaired regulated IκBα degradation and enhanced inhibition of canonical NF-κB activation for the N-terminal variants studied here, they do not establish the full spectrum of receptor-specific NF-κB defects across TNFR, IL-1R, TLR, TCR, or BCR inputs. Future studies using patient-derived cells or physiologically relevant cellular models will be required to assess NF-κB nuclear translocation, IL-1R/TLR-induced cytokine responses, noncanonical NF-κB signaling, antigen-specific humoral immunity, and cell type-specific transcriptional consequences.

In summary, we expand the clinical and molecular spectrum of N-terminal IκBα GOF disease by identifying two novel pathogenic *NFKBIA* variants and defining a reproducible immunologic phenotype characterized by naïve-skewed T-cell and B-cell compartments, a stimulus-dependent T-cell defect in the tested patient, and impaired inducible IκBα degradation. These findings support disturbed lymphocyte maturation and defective regulated IκBα degradation as key features of the N-terminal variants analyzed in this study and provide a framework for earlier diagnosis and future therapeutic stratification.

## Data Availability

The datasets presented in this study can be found in online repositories. The names of the repository/repositories and accession number(s) can be found in the article/**Supplementary Material**.
