# Supplementary material for: De novo NFKBIA variants within the N-terminal hotspot: consistent immunophenotype and divergent clinical presentations
Source: Front Immunol. 2026 Jun 5;17:1854185. doi: 10.3389/fimmu.2026.1854185 (PMC13278860; doi:10.3389/fimmu.2026.1854185)
Supplement: Supplementary file 1 [file Image1.pdf]

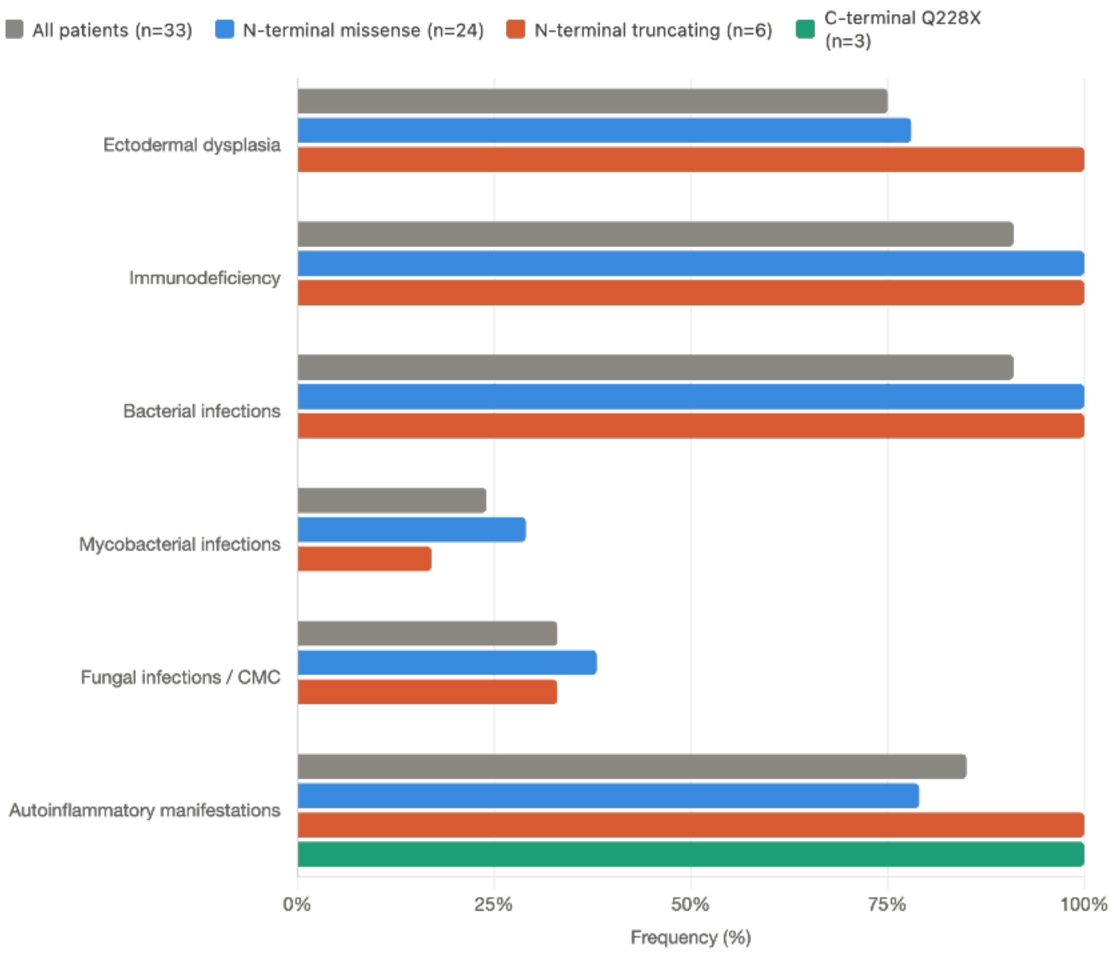

Supplementary Figure S1: Frequency of key clinical features in 33 reported NFKBIA GOF patients, stratified by variant class

Data derived from Supplementary Table S2. For ectodermal dysplasia, one patient with unreported status (P19/D31N) is excluded from the denominator (effective n=32). Percentages are rounded to the nearest integer. CMC, chronic mucocutaneous candidiasis; EDA, ectodermal dysplasia; GOF, gain of function. N-terminal missense: variants within the signal reception domain phosphodegron (D31–S36 and adjacent residues); N-terminal truncating: W11X, Q9X, E14X; C-terminal: Q228X.
