# Supplementary material for: De novo NFKBIA variants within the N-terminal hotspot: consistent immunophenotype and divergent clinical presentations
Source: Front Immunol. 2026 Jun 5;17:1854185. doi: 10.3389/fimmu.2026.1854185 (PMC13278860; doi:10.3389/fimmu.2026.1854185)
Supplement: Supplementary file 2 [file Table1.docx]

**Supplementary Table S1. Immunophenotyping of lymphocytes**

|  | **Percentage % (Reference range [1])** | | | | | | | **Absolute Numbers cells/μL (Reference range [1])** | | | | | | |
| --- | --- | --- | --- | --- | --- | --- | --- | --- | --- | --- | --- | --- | --- | --- |
| **Subject/Age** | **P1** | **Reference** | **P4** | **Reference** | **P2** | **P3** | **Reference** | **P1** | **Reference** | **P4** | **Reference** | **P2** | **P3** | **Reference** |
| **T cells** | **71.7** | 56.84-75.02 | **61.2** | 59.50-75.56 | **88.2↑** | **71.1** | 54.28-71.67 | **2093.4** | 1184-2144 | **1261.0↓** | 1480-2847 | **12689.1↑** | **6768.7↑** | 2179-4424 |
| **CD8+T cells** | **33.9** | 21.91-36.80 | **18.1↓** | 19.70-32.04 | **17.3** | **14.1** | 14.08-24.70 | **990.8** | 489-1009 | **373.8↓** | 552-1127 | **2495.2↑** | **1337.6** | 556-1687 |
| **CD8 Naïve** | **83.7↑** | 35.34-72.32 | **75.0** | 38.03-79.08 | **99.5↑** | **97.5↑** | 68.90-94.60 | **829.3↑** | 231-568 | **255.6↓** | 293-768 | **2482.7↑** | **1304.1↑** | 503-1276 |
| **CD8 TEMRA** | **3.9↓** | 5.08-31.24 | **11.0** | 1.30-22.85 | **0.1** | **0.7** | 0.02-9.61 | **38.1** | 29-269 | **37.5** | 9-209 | **1.8** | **9.5** | 0-133 |
| **CD8 CM** | **9.9↓** | 10.96-31.00 | **10.7↓** | 11.91-36.87 | **0.4↓** | **1.6↓** | 5.14-25.55 | **98.1** | 74-228 | **36.5↓** | 79-350 | **11.0↓** | **21.8↓** | 41-305 |
| **CD8 EM** | **2.6** | 2.38-15.84 | **3.2** | 1.11-14.51 | **0.0↓** | **0.1** | 0.10-4.95 | **25.4** | 16-109 | **10.91** | 7-104 | **0.4↓** | **1.9** | 1-70 |
| **CD4+T cells** | **33.0** | 22.25-39.00 | **39.9** | 28.49-41.07 | **70.4↑** | **55.7↑** | 33.72-52.43 | **962.1** | 522-1084 | **822.2** | 767-1592 | **10124.8↑** | **5300.7↑** | 1461-3018 |
| **CD4 Naïve** | **61.2** | 39.50-66.26 | **73.0↑** | 40.75-72.70 | **97.0↑** | **93.3↑** | 69.15-88.10 | **588.8** | 230-627 | **547.2** | 338-1036 | **9821.1↑** | **4945.6↑** | 1170-2595 |
| **CD4 TEMRA** | **1.5** | 0.00-1.54 | **0.5** | 0.00-1.47 | **0.1** | **0.3** | 0.00-1.64 | **14.5↑** | 0-12 | **4.1** | 0-16 | **5.4** | **18.0** | 0-40 |
| **CD4 CM** | **32.1** | 25.34-49.90 | **24.7** | 21.66-52.74 | **2.7↓** | **5.9↓** | 10.11-28.20 | **308.9** | 182-403 | **185.1↓** | 232-600 | **269.3** | **310.1** | 213-647 |
| **CD4 EM** | **5.2** | 4.68-15.70 | **1.8↓** | 1.90-9.20 | **0.3** | **0.5** | 0.28-2.10 | **50.0** | 29-117 | **13.5↓** | 20-96 | **25.3** | **24.9** | 5-48 |
| **TCRαβ+DNT** | **0.5↓** | 0.61-2.31 | **0.6** | 0.19-2.43 | **0.2↓** | **0.4** | 0.33-1.12 | **9.5↓** | 12-37 | **6.7** | 3-49 | **22.3** | **27.9** | 11-45 |
| **γδ T cell** | **5.7↓** | 6.55-20.28 | **30.7↑** | 7.00-19.60 | **0.5↓** | **2.1↓** | 3.32-7.40 | **118.3** | 81-343 | **352.9** | 133-427 | **68.5↓** | **144.2** | 92-279 |
| **B cells** | **19.7↑** | 8.84-17.76 | **18.5** | 10.46-21.77 | **9.7↓** | **10.1↓** | 17.34-36.03 | **575.2↑** | 203-476 | **380.9** | 303-777 | **1393.0** | **962.5** | 734-2265 |
| **Memory B** | **3.6↓** | 7.15-23.10 | **10.6** | 8.61-20.19 | **0.3↓** | **1.7** | 0.99-4.71 | **20.6** | 20-86 | **36.8↓** | 37-114 | **4.0↓** | **16.2** | 12-54 |
| **Naïve B** | **89.5↑** | 53.78-78.64 | **80.8↑** | 52.04-75.78 | **91.4** | **91.0** | 87.55-94.85 | **514.8↑** | 116-347 | **280.6** | 171-469 | **1273.2** | **875.8** | 691-2132 |
| **Transitional B** | **10.4↑** | 1.38-9.42 | **14.7↑** | 3.41-11.17 | **9.4↓** | **29.2** | 15.05-29.95 | **59.8↑** | 4-37 | **51.0** | 14-59 | **130.2↓** | **281.0** | 136-464 |
| **Plasmablasts B** | **0.8** | 0.49-7.06 | **2.2** | 0.80-9.75 | **0.1↓** | **1.3** | 0.60-3.95 | **4.3** | 1-23.0 | **7.74** | 3-39 | **1.1↓** | **12.0** | 6-42 |
| **NK cells** | **8.6↓** | 10.12-28.34 | **20.2** | 7.83-20.99 | **2.1↓** | **18.7↑** | 5.89-14.85 | **251.4** | 210-804 | **417.0** | 227-667 | **307.9** | **1775.5↑** | 290-780 |
| **CD4/CD8** | **0.97** | 0.65-1.65 | **2.2↑** | 1.02-2.05 | **4.06↑** | **3.96↑** | 1.47-3.23 | **/** | **/** | **/** | **/** | **/** | **/** | **/** |

**CM**, central memory T cells; **EM**, effector memory T cells; **TEMRA**, terminal effector memory T cells re-expressing CD45RA; **DN**, double negative

***[1]*** *Ding Y, Zhou L, Xia Y, et al. Reference values for peripheral blood lymphocyte subsets of healthy children in China. J Allergy Clin Immunol. 2018;142(3):970-973.e8. doi:10.1016/j.jaci.2018.04.022*

***Note****: Patient ages refer to the time of immunophenotyping: P1, 14 years 9 months; P4, 6 years 1 month; P2, 5 months; and P3, 4 months. Published Chinese pediatric reference ranges from Ding et al. were used only as additional reference intervals in this table and were not used to generate the healthy control data points in Figure 2A.*
