# Supplementary material for: De novo NFKBIA variants within the N-terminal hotspot: consistent immunophenotype and divergent clinical presentations
Source: Front Immunol. 2026 Jun 5;17:1854185. doi: 10.3389/fimmu.2026.1854185 (PMC13278860; doi:10.3389/fimmu.2026.1854185)
Supplement: Supplementary file 3 [file Table2.docx]

**Supplementary Table S2. Genetic and clinical features of patients with heterozygous NFKBIA variants.**

| Patient | Gender | Ethnicity | Age of onset | Variant | Inheritance | Infection Manifestations | Autoinflammatory Manifestations | EDA | ID | Growth Development | Other Phenotype | Treatment | Outcome | Ref. |
| --- | --- | --- | --- | --- | --- | --- | --- | --- | --- | --- | --- | --- | --- | --- |
| P1 | M | Italian | 2 months | S32I | De novo | Recurrent LRTI: P. aeruginosa, Klebsiella, Serratia, S. aureus CMC | fever, sterile periostitis, pustular skin, rash, and soft tissue swelling | Yes | Yes | Retardation | chronic diarrhea; hepatosplenomegaly | HSCT IVIG | Alive at 23 years (2019) | Courtois *et al.*, 2003 |
| P2 | M | Caucasian | 2 years | S32I mosaicism | De novo | enteritis (Salmonella typhimurium) S. typhimurium infection persisted with recurrent manifestations in psoas muscle, pleural cavity, pericardial fluid, and ribs | fever, typical rash, and JIA | No | Yes | No | chronic diarrhea | nr | Alive at > 20 years (2004) | Janssen *et al*., 2004 |
| P3 | M | Caucasian | 2 months | S32I | Inherited from his father | Meningitis: β-hemolytic group A Streptococcus: sepsis Respiratory infection pneumonia Pneumocystis jirovecii, mild CMC | fever | Yes | Yes | Retardation | chronic diarrhea | HSCT antibiotics cotrimoxazole | Dead (2005) | Janssen *et al*., 2004 |
| P4 | F | American | Birth | W11X | Mother: WT; Father: nr | Recurrent pneumonia | fever, skin rash, | Yes | Yes | nr | nr | IVIG corticosteroid antibiotics | Alive at 22 years (2016) | McDonald *et al.*, 2007 |
| P5 | M | Caucasian | 1 month | E14X | De novo | Pneumonia (parainfluenza virus and Pneumocystis carinii), recurrent episodes of bacteremia oral candidiasis. Pyogenic bacteria sepsis, CMC | fever | Yes | Yes | Retardation | feeding intolerance, frequent episodes of diarrhea | HSCT trimethoprim- sulfamethoxazole | Dead (1 year, 2008) | Lopez-Granados *et al*., 2007 |
| P6 | M | Japanese | 1 month | Q9X | nr | Bacterial: pneumonia, respiratory syncytial virus. Bronchiolitis, acute otitis media, urinary tract infection. Cytomegalovirus: hepatitis, Rotavirus: enteritis. Bronchiolitis with respiratory syncytial virus | systemic inflammation | Yes | Yes | nr | chronic diarrhea | HSCT | Alive at 7 years (2016) | [Ohnishi](https://pubmed.ncbi.nlm.nih.gov/?term=Ohnishi+H&cauthor_id=22078572) *et al.*, 2012 |
| P7 | M | Japanese | 4 months | S36Y | De novo | BCG skin infection | systemic inflammation | Yes | Yes | Retardation | Gastroenteritis, chronic diarrhea | HSCT | Dead (2013) | Yoshioka *et al.*, 2013 |
| P8 | M | Caucasian | 6 months | M37K | Mother: WT  Father: nr | Haemophilus influenza: pneumonia, CMC | fever | Yes | Yes | Retardation | chronic diarrhea | HSCT | Dead (2013) | Schimke *et al.*, 2013 |
| P9 | F | Italian | 5 months | M37R | nr | Recurrent LRTI Sepsis: Klebsiella pneumonia, Candida parapsilosis, Stenotrophomonas maltophilia, osteomyelitis of skull and limb, CMC | fever | Yes | Yes | nr | chronic diarrhea | nr | Dead (2013) | Giancane *et al*., 2013 |
| P10 | F | Chinese | 1 month | S36Y | De novo | LRTI: P. aeruginosa: bronchiectasis and sinusitis Mycobacterium tuberculosis: abdominal lymphadenopathy M. abscessus: septic arthritis of the knee, osteomyelitis Urinary tract infection: K. pneumoniae | fever | No | Yes | nr | nr | Subcutaneous rIFN-γ (50 μg/m2), cotrimoxazole, IVIG, antituberculosis treatment | Alive at 9 years (2015) | Lee *et al*., 2016 |
| P11 | F | Caucasian / Thai | 20 months | S32G | De novo | Salmonella enteritidis: osteomyelitis, hematochezia Candida: esophagitis Mycobacterium malmoense: blood and skin Sapovirus and norovirus: stool | fever | Yes | Yes | No | chronic diarrhea | HSCT IVIG | Alive at 6 years (2017) | Staples *et al.*, 2017 |
| P12 | M | Japanese | 2 months | S32R | De novo | S. aureus sepsis, CMC, Recurrent pneumonia | fever, skin erythema, systemic inflammation | Yes | Yes | nr | nr | HSCT | Dead at 2 years | Moriya *et al.*, 2018 |
| P13 | M | Japanese | Birth | S32N | De novo | S. aureus and P. aeruginosa sepsis | fever | Yes | Yes | nr | bloody stool, inflammatory bowel disease; recurrent intracranial hemorrhage; difficulty in hemostasis | HSCT | Dead at 1.5 years | Moriya *et al.*, 2018 |
| P14 | nr | nr | nr | S32I | ? | Recurrent infections | fever | Yes | Yes | nr | nr | HSCT IVIG | Alive at 10 years (2017) | Petersheim *et al*., 2017 |
| P15 | nr | nr | nr | G33V | ? | Recurrent infections | fever | Yes | Yes | nr | nr | HSCT IVIG | Alive at 10 years (2017) | Petersheim *et al*., 2017 |
| P16 | M | Turkish | early childhood | S36A | De novo | Recurrent gastrointestinal infections (Shigellosis, C. jejuni), recurrent upper respiratory tract infections (bronchitis, sinusitis, otitis media), recurrent pneumonias (S. pneumoniae, H. influenzae), bronchiectasis with chronic mucoid Pseudomonas aeruginosa infection, meningitis (N. meningitidis), CNS tuberculosis with brain abscess, verruca vulgaris (HPV 9 and 57) | fever, JRA | No | Yes | No | Warts phimosis | IVIG chloroquine methotrexate azathioprine azithromycin prophylaxis gentamycin | Alive at 37 years (2019) | Sogkas *et al.*, 2020 |
| P17 | F | Turkish | early childhood | S36A | Inherited from her father | Recurrent upper respiratory tract infections (bronchitis, otitis media), recurrent pneumonias, bronchiectasis, Verruca vulgaris | No | No | Yes | No | No | Co-trimoxazole prophylaxis | Alive at 9 years (2019) | Sogkas *et al.*, 2020 |
| P18 | F | Turkish | early childhood | S36A | Inherited from her father | Recurrent upper respiratory tract infections (bronchitis, otitis media), two pneumonias within two years | No | No | Yes | No | Warts | Co-trimoxazole prophylaxis | Alive at 4 years (2019) | Sogkas *et al.*, 2020 |
| P19 | M | Spain | Birth | D31N | De novo | DIRA-like phenotype (pustular skin, periostitis, systemic inflammation) | fever Pustular skin rash soft tissue swelling | nr | Yes | nr | Sterile periostitis Multiorgan failure | nr | Dead soon after birth | Batlle-Masó et al., 2020 |
| P20 | F | nr | 1 week | L34P | De novo | Disseminated BCG infection with abscess; recurrent severe pneumonias; invasive bacterial/fungal infections;rotavirus gastroenteritis | IL-1β hypersecretion / activated neutrophilia; liver inflammation with fibrosis/cholestasis; psoriasiform rash | Yes | Yes | nr | hepatosplenomegaly; pulmonary alveolar proteinosis | antimicrobials; HSCT; anakinra | Alive at 25.9 months / alive at last follow-up in 2020 | Tan et al., 2020 |
| P21 | F | Chinese | 2 months | E14X | De novo | Recurrent bronchopneumonitis (Gram ± pyonenic) | fever | Yes | Yes | Retardation | bloody stool, chronic diarrhea; hepatosplenomegaly | IVIG antibiotics | Dead (2020) | Wen et al., 2022 |
| P22 | M | Malaysia | Birth | S32C | De novo | Congenital pneumonia; persistent oral/perianal thrush; recurrent severe pneumonia; S. aureus, K. pneumoniae, Enterobacter and Salmonella sepsis; melioidosis; possible pulmonary aspergillosis | No | Yes | Yes | nr | hypothyroidism; autoimmune hemolytic anemia; thrombocytopenia; | IVIG; co-trimoxazole; itraconazole; L-thyroxine | Dead at 17 months (2022) | Chear et al., 2022 |
| P23 | M | Syrian | 8 months | W11X | De novo | post-BCG lupus vulgaris / cutaneous Mycobacterium bovis infection with right axillary abscessed adenopathy; no other infection history reported | recurrent fever of unknown origin | Yes | Yes | nr | left knee synovitis | isoniazid + rifampicin + ethambutol for 3 months, then isoniazid + rifampicin to a total of 12 months | Alive, asymptomatic without prophylaxis (2023) | Tapiero et al., 2023 |
| P24 | M | American | Birth | D31N | De novo | Recurrent MSSA and Serratia marcescens bacteremia; recurrent Serratia pneumonia | persistent erythematous rash; temperature dysregulation | Yes | Yes | Intrauterine growth restriction | thrombocytopenia; micrognathia, ear cupping, accessory ribs, cardiac calcifications; encephalomalacia/seizures; rickets | fluconazole; co-trimoxazole / TMP-SMX; IVIG; azithromycin prophylaxis; HSCT | Dead after HSCT (2023) | Gunderman et al., 2023 |
| P25 | M | Chinese | 6 months | Q9X | De novo | Bronchopneumonia; recurrent diarrhea/colitis with Candida and enterobacteria; urethritis; influenza A infection | fever; colonic ulcer, rectal ulcer, ulcerative stomatitis | Yes | Yes | nr | lymphocytosis; increased NK and CD8+ T cells | IVIG; itraconazole; co-trimoxazole prophylaxis | Alive, awaiting HSCT (2024) | Wu et al., 2024 |
| P26 | M | German | 20 years | Q228X | nr | none reported | seronegative chronic polyarthritis | No | No | No | atopic dermatitis; laryngeal cancer; lung cancer | nr | Dead at 74 years (2025) | Elsayed et al., 2025 |
| P27 | M | German | 18 years | Q228X | Inherited from his father | none reported | psoriatic arthritis (initially seronegative erosive chronic polyarthritis); psoriasis vulgaris | No | No | No | dyshidrotic eczema; vocal cord polyps; secondary hypogammaglobulinaemia; reduced switched B cells | corticosteroids; multiple DMARDs; tocilizumab | Alive (2025) | Elsayed et al., 2025 |
| P28 | F | German | 29 years | Q228X | Inherited from her father | none reported | psoriatic arthritis; intermittent arthralgia since late childhood; enthesitis; plantar fasciitis | No | No | No | reduced switched B cells | nr | Alive (2025) | Elsayed et al., 2025 |
| P29 | F | South Slavic | 1 month | S36P | nr | recurrent pneumonias (including P. jirovecii pneumonia); S. hominis hominis (catheter-associated) | No | Yes | Yes | No | pericardial effusion; hepatomegaly; hemangioma; atrial septal defect | HSCT | Dead at 148 days (2025) | Elsayed et al., 2025 |
| P30 | M | Chinese | 12 years | G33D | De novo | Streptococcus pneumoniae pneumonia, otitis media | recurrent fever | Yes | Yes | No | chronic bronchitis and bronchiectasis | IVIG, antibiotics. | Alive (2026) | **(this study)** |
| P31 | M | Chinese | 1 month | M37R | De novo | Local BCGitis, Pseudomonas aeruginosa, Pneumocystis jirovecii pneumonia, CMC | recurrent fever | Yes | Yes | No | diarrhea with hematochezia, anemia ,hepatosplenomegaly | IVIG, antibiotics and antimycobacterial regimen. | Died of ARDS at 9 months | **(this study)** |
| P32 | M | Chinese | 2 months | M37K | De novo | Local BCGitis, Streptococcus pneumoniae pneumonia, Rotavirus, CMC | No | Yes | Yes | No | chronic bronchitis and bronchiectasis, lymphadenectasis, anemia | IVIG, antibiotics and antimycobacterial regimen. HSCT at 1.5 years | Alive (2026) | **(this study)** |
| P33 | F | Chinese | 2 months | D31H | De novo | Local BCGitis, pneumonia | recurrent fever | Yes | Yes | No | anemia | IVIG, antibiotics and antimycobacterial regimen. | Alive (2026) | **(this study)** |

***LRTI****, Lower Respiratory Tract Infection;* ***JRA****, juvenile rheumatoid arthritis;* ***JIA****, juvenile idiopathic arthritis;* ***CMC****, chronic mucocutaneous candidiasis;* ***IVIG****, intravenous immunoglobulin;* ***HSCT****, hematopoietic stem cell transplantation;* ***nr****, not report; P2 is father of P3; P16 is father of P17 and P18; P26 is father of P27 and P28*
