# Supplementary material for: De novo NFKBIA variants within the N-terminal hotspot: consistent immunophenotype and divergent clinical presentations
Source: Front Immunol. 2026 Jun 5;17:1854185. doi: 10.3389/fimmu.2026.1854185 (PMC13278860; doi:10.3389/fimmu.2026.1854185)
Supplement: Supplementary file 4 [file Table3.docx]

**Supplementary Table S3. Molecular and cellular phenotypes of patients with heterozygous NFKBIA variants.**

| Patient | IκBα degradation (agonist-cell type) | NF-κB translocation (agonist-cells) | IL-1R/TLR pathway activation (agonist-cell type) | TNFR pathway activation (agonist-cell type) | T-cell response in PBMCs (stimulus) | B-cell prolif (stimulus) |
| --- | --- | --- | --- | --- | --- | --- |
| P1-S32I | Impaired (TNF-α, LPS-fibroblast) | Impaired (p50/ p65; p50/p50- TNF-α- fibroblast) | Impaired (LPS-PBMC) Impaired (LPS,IL-1β- fibroblast) | Impaired (TNF- α; LTα1β2- fibroblast) | Absent prolif. (low α- CD3, recall Ags) Normal prolif. (α- CD3/α-CD28, PMA, allogeneic cells) Normal IFN-γ prod. (α-CD3, α-CD3/α- CD28) | nr |
| P2-S32I mosaicism | nr | nr | Impaired (LPS, PAM3, zymosan-WB) Impaired (LPS-MdM) Impaired (LPS- fibroblast) | Impaired (TNF- α; LTα1β2- fibroblast) | Low prolif. (low α- CD3, PHA) Normal prolif | Normal (CD40L + IL4) |
| P3-S32I | Impaired (LPS-fibroblast) | Impaired (p65- LPS-MdM) | Impaired (LPS, PAM3, zymosan-WB) Impaired (LPS-MdM) Impaired (LPS- fibroblast) | nr | Low prolif. (low α- CD3, PHA) Absence prolif. (recall Ags) | Normal (CD40L + IL4) |
| P4-W11X | Impaired (LPS-fibroblast) | Impaired (p50/ p65-IL-1β- fibroblast) | Impaired (IL-1β, LPS- fibroblast) Impaired (poly(I:C), LPS, flagellin, CpG-PBMC) | nr | Normal prolif. (low α-CD3, α-CD3/α- CD28, PMA/iono, PHA, recall antigens) | nr |
| P5-E14X | Impaired (CD40L-EBV-B) | Impaired (p50; p65; c-Rel- CD40L-EBV-B) | Impaired (LPS, SAC OspA-PBMC) | Impaired (CD40L-EBV B cells) | Normal prolif. (PHA, ConA, and recall Ags) Impaired IFN-γ and TNF-α prod. (α-CD3) | nr |
| P6-Q9X | nr | nr | Impaired (LPS-monocyte) Impaired (LPS-fibroblast) | nr | Low prolif. (PHA; ConA) | nr |
| P7-S36Y | Impaired (TNF-α-T blast cells) | Impaired (p50/ p65-TNF-α- fibroblast) | Impaired (LPS, IL-18 -PBMC) | Impaired (TNF- α, LTα1β2- fibroblast) Impaired (CD40L-PBMC) | Low prolif. (low dose of α-CD3), Normal prolif. (high dose of α-CD3), Normal prolif. (PHA; PMA, recall Ags) | nr |
| P8-M37K | Impaired (TNF-α, LPS, PAM3-fibroblast) | Impaired (p50/ p65-TNF-α- HeLa cells) | Impaired (LPS, PAM3- fibroblasts) Normal (SAC-WB); impaired (IL-1β, SAC, LPS, PAM2, PMA/Iono- WB) | Impaired (TNF- α-fibroblast) Impaired (TNF- α-WB) | Low prolif. (OKT3, SAC), Normal prolif. (PHA, PWM, ConA, recall Ags, diphtheria, tetanus/ streptolysin O/ mumps) | nr |
| P9-M37R | nr | nr | nr | nr | Normal prolif. (PHA, PMA, α-CD3/α-CD28) | Decreased (CpG) |
| P10-S36Y | Impaired (TNF-α, IL-1β-fibroblast) | nr | Impaired (IL-1β -fibroblast) | Impaired (TNF- α-fibroblast) | Impaired prolif. (high α-CD3) Normal prolif. α-CD3/α-CD28, PHA, ConA, PMA/iono Impaired (IFN-γ and IL-12 production; BCG, BCG/IL-12; BCG/IFN-γ) | nr |
| P11-S32G | Impaired (TNF-α-fibroblasts) | nr | Impaired (LPS-WB) | nr | Normal prolif. (PHA) | nr |
| P12-S32R | Impaired (TNF-α-fibroblasts) | nr | nr | nr | nr | nr |
| P13-S32N | Impaired (CD40L-EBV-B) | nr | nr | nr | nr | nr |
| P14-S32I | nr | nr | nr | nr | nr | nr |
| P15-G33V | Impaired (LPS-fibroblasts) | nr | Impaired (LPS -fibroblast) | nr | nr | nr |
| P16-S36A | Impaired (PMA/iono, anti-IgM and LPS-B cell) | nr | nr | nr | nr | nr |
| P17-S36A | nr | nr | nr | nr | nr | nr |
| P18-S36A | nr | nr | nr | nr | nr | nr |
| P19-D31N | nr | nr | nr | nr | nr | nr |
| P20-L34P | Impaired (TNF-α, LPS-fibroblast) | Impaired (p65-TNF-α/LPS-fibroblasts) | Impaired (IL-1β, LPS-fibroblast) | Impaired (TNF-α- fibroblast) | Poor proliferation to PHA and Con-A | nr |
| P21-E14X | nr | Impaired (p65- LPS-PBMC) | Impaired (LPS,IL-1β- PBMC) | nr | nr | nr |
| P22-S32C | nr | nr | nr | nr | nr | nr |
| P23-W11X | nr | nr | nr | nr | nr | nr |
| P24-D31N | Impaired (IL-1β- fibroblast) | nr | Impaired (IL-1β- fibroblast) | Impaired (TNF, TNFβ- fibroblast) | Near absent lymphocyte proliferation to mitogens PHA and PWM | nr |
| P25-Q9X | nr | nr | Altered cytokine response (LPS-PBMC) | Impaired NF-κB reporter (TNF-α-HEK293T) | Defective proliferation to α-CD3 (low dose) and PHA | nr |
| P26-Q228X | nr | nr | nr | nr | nr | nr |
| P27-Q228X | Normal (PMA-T; anti-IgM-B; LPS-monocyte) | Impaired (p50- PMA/iono-PBMC) | Enhanced IL-1β secretion (LPS+ATP- monocyte) | nr | nr | nr |
| P28-Q228X | Normal (PMA-T; anti-IgM-B; LPS-monocyte) | Impaired (p50- PMA/iono-PBMC) | Enhanced IL-1β secretion (LPS+ATP- monocyte) | nr | nr | nr |
| P29-S36P | Impaired/abrogated (PMA-T; anti-IgM-B; LPS-monocyte) | nr | nr | nr | nr | nr |
| P30-G33D (this study) | Impaired (TNF-α, PBMCs) | nd | nd | Impaired (TNF-α, PBMCs) Impaired NF-κB reporter (TNF-α-HEK293T) | Normal prolif. (PHA) | Normal (CpG + anti-IgMF(ab')₂ |
| P31-M37R (this study) | Impaired (TNF-α, PBMCs) | nd | nd | Impaired (TNF-α, PBMCs) Impaired NF-κB reporter (TNF-α-HEK293T) | nd | nd |
| P32-M37K (this study) | Impaired (TNF-α, PBMCs) | nd | nd | Impaired (TNF-α, PBMCs) Impaired NF-κB reporter (TNF-α-HEK293T) | Normal prolif. (PHA) Low prolif. (α- CD3/α-CD28) | Normal (CpG + anti-IgMF(ab')₂ |
| P33-D31H (this study) | Impaired (TNF-α, PBMCs) | nd | nd | Impaired (TNF-α, PBMCs) Impaired NF-κB reporter (TNF-α-HEK293T) | nd | nd |

*nr, not reported; nd, not detected; WB, whole blood; MdM, macrophage-derived monocytes; Ags, antigens; Prolif, proliferation; PWM, Pokeweed mitogen; Con-A, Concanavalin-A; PHA, Phytohemagglutinin; PMA, Phorbol 12-myristate 13-acetate.*
